# Vitreous induces heme oxygenase-1 expression mediated by transforming growth factor-β and reactive oxygen species generation in human retinal pigment epithelial cells

**Published:** 2007-01-24

**Authors:** Ren Hartung, Sunil K. Parapuram, Ramapriya Ganti, D. Margaret Hunt, Kakarla V. Chalam, Richard C. Hunt

**Affiliations:** 1 Department of Pathology and Microbiology, University of South Carolina School of Medicine, Columbia, SC; 2 Department of Ophthalmology, University of Florida at Jacksonville, Jacksonville, FL

## Abstract

**Purpose:**

When human retinal pigment epithelial (RPE) cells come in contact with vitreous, they undergo changes in gene expression that include inflammatory and anti-oxidant responses. The effects of vitreous on expression of heme oxygenase-1 (HO-1), metallothionein (MT) -1a and -2a, and c-fos were investigated. Activator protein-1 (AP-1) binding sites are located in the promoter region of HO-1 and MT genes and the effects of vitreous on c-fos activity were investigated.

**Methods:**

Low passage cultures of human RPE cells were grown in the presence or absence of vitreous or transforming growth factor-β (TGF-β). The expression of HO-1 and MTs was measured by real time PCR and, in the case of HO-1, by immunoblotting and immunofluorescence microscopy. Specific inhibitors were used to investigate possible signaling pathways. The effect of vitreous on activation of AP-1 transcription factor was determined by immunoblotting, electrophoretic mobility shift assays, or immunofluorescence microscopy.

**Results:**

Incubation of RPE cells with vitreous resulted in increased expression of HO-1, MT-1a and MT-2a. TGF-β caused an increase in HO-1 expression, although not to the extent mediated by vitreous, but had little effect on MT expression. Addition of inhibitors of TGF-β signaling (SB431542 or TGF-β-neutralizing antibodies) decreased the vitreous induction of HO-1. Several reactive oxygen species (ROS) quenchers inhibited the TGF-β-induced or vitreous-induced elevation of HO-1 mRNA but had no effect on vitreous-mediated induction of MT expression. Inhibitors of the mitogen-activated protein kinase (p38MAPK; SB203580) and Jun N-terminal kinase (JNK; SP600125) pathways inhibited vitreous-induction of HO-1. C-fos, a component of AP-1 transcription factor complexes, exhibited increased expression and activation in the presence of vitreous.

**Conclusions:**

TGF-β, a known component of vitreous, can account for some but not all of the regulation of the anti-oxidant, anti-inflammatory HO-1 gene in human RPE cells, but it does not participate in the vitreous-mediated upregulation of MTs. Both vitreous and TGF-β signals increased HO-1 expression via ROS but the latter were not involved in vitreous-mediated MT expression. Increased p38, JNK, and c-fos activation may be implicated in vitreous modulation of HO-1.

## Introduction

Retinal pigment epithelial (RPE) cells form a monolayer between the retina and the choriocapillaris. These cells constitute one aspect of the blood retinal barrier and play a critical role in the maintenance of the neural retina [1]. They do not normally divide after birth but may do so in some pathological situations. In proliferative vitreoretinopathy (PVR), for example, breach of the blood-retinal barrier, accompanied by a tear in the neural retina that allows vitreous contact with the RPE cell monolayer, can lead to cell division and epithelial-mesenchymal transformation (EMT) of the RPE cells. The resulting fibroblast-like cells can move into the vitreous where they participate in the formation of a fibrotic epiretinal membrane that may contract, leading to retinal detachment [2]. Risk factors for PVR include trauma to the eye, contact between RPE cells and the vitreous, breakdown of the blood-retinal barrier, and inflammation [3-5]. Gene array analyses of the changes that occur in cultured RPE cells that have been exposed to vitreous and undergo EMT indicate an inflammatory or stress response as the cells transform [6] (Ganti et al. Investigative Ophthalmology and Visual Science. In press). The vitreous-induced changes in gene expression also include increased expression of genes involved in anti-oxidant responses, such as heme oxygenase-1 (HO-1), metallothioneins (MT), and hypoxia-induced factor-1 (Ganti et al., In press). Increased expression of such genes may help to resolve inflammation and protect the cells from apoptosis.

HO-1 participates in many anti-inflammatory, anti-oxidant and anti-apoptotic responses [7] and is expressed by human RPE [8-12] and other [7] cells under a variety of conditions. For example, HO-1 expression is increased by such stimuli as heavy metals, hypoxia, hyperoxia, inflammation, and certain growth factors and cytokines, many of which signal via reactive oxygen species (ROS) generation [7]. MTs are small proteins containing up to 30% cysteine which bind metals, particularly zinc, and are powerful anti-oxidants that may participate in the resolution of inflammation [13]. Since MTs, like HO-1, are induced in response to oxidative stress and inflammation, it is not surprising that many of the same factors control their expression [7,14]. Transforming growth factor-β (TGF-β) is a growth factor involved in EMT, cell migration, proliferation, and apoptosis during normal development and in certain diseases, including PVR [15]. It is present in normal vitreous [15] and increases HO-1 expression in human RPE cells [8].

We investigated the vitreous induction of HO-1 and MT in low passage human RPE cells. We found that vitreous led to an increase in HO-1 expression that was partly caused by TGF-β, and that the rise in HO-1 expression was signaled via ROS generation. MT-1a and MT-2a were also induced by vitreous but their expression was not under the control of TGF-β or ROS. Concurrent with these changes in anti-oxidant proteins was the activation of c-fos, a component of AP-1 transcription factor complexes that binds to sites in the promoters of HO-1 [7,16-18], MT-1 [19] and MT-2a [20] genes and which has been implicated in anti-oxidant and anti-inflammatory responses.

## Methods

### Human vitreous and retinal pigment epithelial cells

Human donor eyes were obtained postmortem from Lifepoint (Columbia, SC) or The Lions' Eye Bank (Portland, OR). The protocol adhered to the tenets of the Declaration of Helsinki for research involving human tissue. The cornea, iris and lens were removed and the vitreous obtained by inverting the eyecup and gently squeezing the eye while pulling the vitreous gel out into a sterile plastic culture dish. RPE cells were isolated as described previously [21] and cultured in F-10 medium (Invitrogen, Carlsbad, CA) containing 10% fetal bovine serum (Invitrogen), 1% penicillin-streptomycin-glutamine (Invitrogen), 1% CaCl_2_ and 1% ITS culture supplement (BD Biosciences, San Diego, CA). The RPE cells were used at passages 3-7 when their morphology remained epithelioid. The epithelial nature of the cells was confirmed by staining for keratin [22-24].

### Treatment with vitreous

The vitreous gel was shredded using a syringe, diluted by adding 3 parts of the above culture medium to 1 part of vitreous and filtered using a 0.22 μm polyethersulfone filter bottle (Corning, Corning, NY). The medium was removed from subconfluent RPE cells that were then treated with 25% vitreous in complete medium, or fresh complete medium, for various times. The cells were subconfluent at the time of treatment and were still subconfluent at the time of RNA or nuclear protein extraction.

In some experiments, medium or vitreous-containing medium was supplemented with TGF-β1 (R&D Systems, Minneapolis, MN), SB431542, a TGF-beta type I receptor antagonist [25] (Tocris Bioscience, Ellisville, MO), neutralizing pan-specific anti-TGF-β antibody (R&D Systems). ROS generation was inhibited by N-acetyl cysteine (Sigma-Aldrich, St Louis, MO), catalase (Sigma-Aldrich), taurine (Sigma-Aldrich) or dimethyl sulfoxide (Sigma-Aldrich). The extracellular signal-regulated kinases (ERK), c-jun-N-terminal kinases (JNK), p38 MAPK and phosphatidylinositol 3'-kinase (PI3) kinase were inhibited by PD98059 (Calbiochem, San Diego, CA), SP600125 (Sigma-Aldrich), SB203580 (Calbiochem), and LY294002 (Sigma-Aldrich), respectively.

### RNA extraction, reverse transcription, and real-time quantitative PCR

RNA was extracted using the RNeasy kit (Qiagen, Valencia, CA) and treated with DNase on the column. Reverse transcription was carried out with 1 μg total RNA per 20 μl reaction using the iScript cDNA Synthesis Kit (Bio-Rad, Hercules, CA) and simultaneous priming with both random hexamers and oligo (dT); Ganti et al., In press. After reverse transcription, the reactions were diluted to 300 μl. "Negative-control" reactions without reverse transcriptase were carried out to ensure that the results were RNA dependent. Real-time quantitative PCR (qPCR) was performed in 25 μl reactions using iQ SYBR Green Supermix (Biorad) with 1 μM of the appropriate primers, and 5 μl of diluted cDNA The PCR products were detected using an IQ Real Time PCR iCycler (Bio-Rad). PCR primers were designed using Oligo 6 (Molecular Biology Insights, Cascade, CO) and are listed in Table 1. Expression of ribosomal protein, large, P0 (RPLP0) mRNA was used as an internal standard (normalization/loading control). The ratio of target gene mRNA expression in treated versus untreated cells was determined according to the method of Pfaffl et al. using the Real-time Expression Software Tool for Excel (REST-XL) version 2 [26,27]. This method determines the changes in the target gene expression relative to changes in the internal standard (RPLP0) and corrects for differences between the efficiencies of the different primer pairs (for efficiencies, see Table 1). The software applies a randomization test (2000 randomizations) to determine statistical significance of the results. Use of REST-XL analysis in the non-normalized mode showed that levels of RPLP0 gene expression did not vary significantly between control and vitreous-treated cells. Each qPCR assay was performed in quintuplicate, and the experiment was repeated at least three times with different RPE cell/vitreous donors.

**Table 1:** Primers used for _Q_PCR.

**Gene**	**Primers (5'-3')**
Heme oxygenase 1 (HO-1)	F: GTCCGCAACCCGACAGCA
	R: TCCTCCAGGGCCACATAGATG
Metallothionein 1a (MT1a)	F: GCGCCTTATAGCCTCTCAAC
	R: CAAGTTTGTGCAGGTCACTCT
Metallothionein 2a (MT2a)	F: ACTCTAGCCGCCTCTTCAG
	R: AAGTCGCGTTCTTTACATCTG
Normalization gene: Ribosomal protein, large, PO (RPLPO)	F: TTAAACCCCCTCGTGGCAATC
	R: CCACATTCCCCCGGATATGA

F represents forward primer; R represents reverse primer.

### Western blotting

Cells were washed with ice cold PBS and lysed with a protein extraction buffer (25 mM HEPES pH 7.5, 100 mM NaCl, 10% glycerol, 1% Triton X100, 50 mM NaF, 1 μM sodium metavanadate, 20 μl protease inhibitor cocktail/ml [Pierce]). SDS-PAGE was carried out using 4-15% acrylamide Criterion pre-cast gels (Bio-Rad) and blotted onto Immun-Blot PVDF membranes (Bio-Rad). Antibodies used were: rabbit polyclonal anti-HO-1 (Stressgen, Ann Arbor, MI; SPA-896), goat polyclonal anti-tubulin (Santa Cruz Biotechnology, Santa Cruz, CA; SC-9935), and rabbit polyclonal anti-human c-Fos (Santa Cruz; SC7202x). Alkaline phosphatase-conjugated secondary antibodies were used together with CDP Star chemiluminescence detection reagent (Perkin Elmer Life Science, Boston, MA).

### Immunofluorescence microscopy

RPE cells were grown on sterile glass cover slips in six-well plates. They were washed with ice cold PBS, fixed with 4% paraformaldehyde, quenched with 10 mM NH_4_Cl in PBS, permeabilized with 0.1% Triton X100 in PBS, incubated with primary antibody in PBS with 5 mg BSA/ml overnight at 4 °C. The cells were then washed with PBS/BSA, incubated with Oregon Green-488 conjugated anti-rabbit IgG secondary antibody or a Texas-Red anti-goat IgG secondary antibody (Molecular Probes, Eugene, OR) for 1 h at 37 °C, and imaged using a Zeiss Metaphor confocal fluorescence microscope.

### Nuclear extracts

Nuclear extracts were made using a kit according to the manufacturer's instructions (Active Motif, Carlsbad, CA). For experiments involving phosphatase treatment,we prepared nuclear protein extracts using a kit that contained phosphatase inhibitor-free solutions (Panomics, Freemont, CA).

### Electrophoretic mobility shift assay

Electrophoretic mobility shift assays (EMSA) were carried out using EMSA "Gel-Shift" (Panomics) according to the manufacturer's instructions. Nuclear extracts were incubated with a biotin-labeled oligonucleotide containing the consensus binding sequence for AP-1 (5'-CGC TTG ATG ACT CAG CCG GAA-3') for 30 min at 15 °C, and transcription factor-bound oligonucleotide was separated from unbound oligonucleotide by electrophoresis on a 5% polyacrylamide gel. After transfer to Biodyne B nylon membrane (Pall Corporation, Ann Arbor, MI) using a semi-dry blot apparatus (Bio-Rad), the biotin-labeled bands were visualized using horseradish peroxidase-based chemiluminescence. The image was captured with a Kodak Image station 440 CF. The specificity of binding was verified using unlabeled consensus oligonucleotide corresponding to AP-1 binding sequence as a competitor in the binding reaction. To verify further the identity of the transcription factor bound to the oligonucleotide, supershift assays were carried out using polyclonal rabbit anti-human c-fos (TransCruz; sc-7202x, Santa Cruz). The reaction mixture was incubated for 1 h at 20 °C with 2 μl of the antibody.

## Results

### Cultured human retinal pigment epithelial cells increase expression of heme oxygenase-1, metallothionein-1, and -2 when exposed to vitreous

In previous work, cultured human RPE cells were exposed to 25% vitreous in serum-containing medium for various periods of time up to 48 h and mRNA expression was examined using gene arrays (21,318 genes) for three independent RPE donor/vitreous donor pairs (Ganti et al., In press). Genes associated with inflammation and stress, including interleukin-1β (IL-1β), microsomal prostaglandin E synthase (mPGES), and cycloöxygenase-2 (COX-2) were significantly overrepresented among the genes induced by vitreous compared to their representation in the total genes on the arrays. Vitreous treatment also resulted in increased expression of mRNAs encoding a number of anti-oxidant proteins, including HO-1 and MT-1E. We have previously shown that human RPE cells express MT-1a and MT-2a [14]; however, neither of these genes was represented on the microarrays.

**Figure 1:**
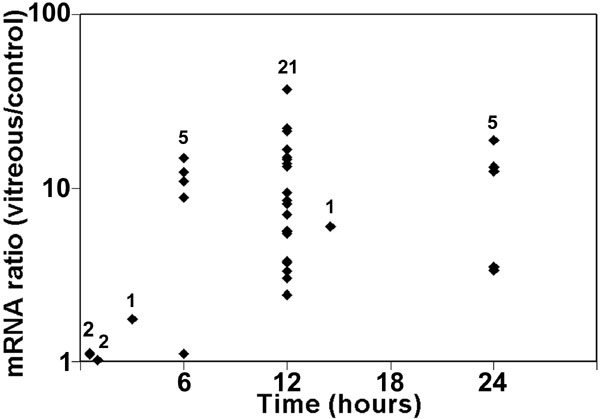
Expression of heme oxygenase-1 mRNA in vitreous-treated retinal pigment epithelial cells. Low passage donor retinal pigment epithelial (RPE) cells were incubated with or without vitreous for up to 24 h. RNA was extracted at each time point and heme oxygenase-1 (HO-1) mRNA was measured by qPCR. Ribosomal protein, large, P0 (RPLP0) was used as an internal standard to correct for small differences in the amount of cDNA used in each reaction. Each point at a particular time indicates a different RPE/vitreous donor pair. The number of donor/vitreous pairs examined at each time point is shown. At 6, 12, and 24 h, REST-XL analysis indicated that HO-1 mRNA was significantly increased (p<0.05) in vitreous-treated RPE cells compared to cells in vitreous-free medium.

**Figure 2:**
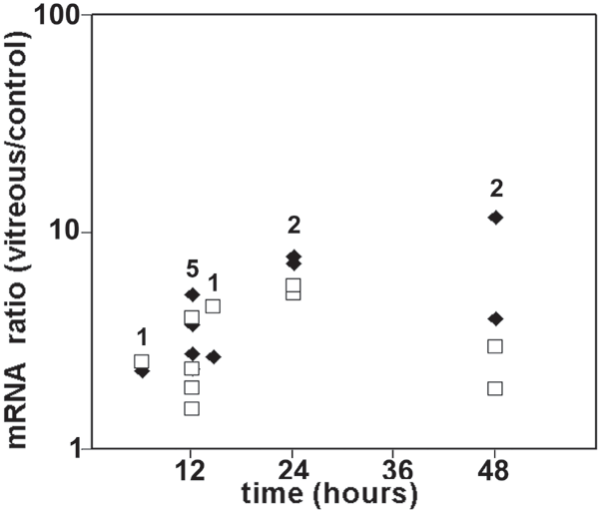
Expression of metallothionein mRNAs in vitreous-treated retinal pigment epithelial cells. Retinal pigment epithelial (RPE) cells were incubated with or without vitreous for up to 48 h. RNA was extracted at each time point and RNAs for metallothionein-1a (MT-1a; quadrangle) and metallothionein-2a (MT-2a; diamond) were measured by qPCR. Ribosomal protein, large, P0 (RPLP0) was used as an internal standard to control for small differences in the amount of cDNA used in each reaction. Each point at a particular treatment time indicates a different RPE/vitreous donor pair. The number of donor/vitreous pairs examined at each time point is shown. At all time points, REST-XL analysis indicated that MT-1 and MT-2a mRNAs were significantly increased (p<0.01) in vitreous-treated RPE cells compared to cells in vitreous-free medium.

Changes in mRNA expression of some anti-oxidant genes were examined by qPCR. Multiple RPE cell and vitreous donors were used in these experiments. HO-1 mRNA expression rose by 3 h after contact with vitreous and reached a plateau after 6 h with a mean increase of approximately 11 fold over the control cells in normal medium without vitreous (Figure 1). Expression of MT-1a and MT-2a mRNAs was also increased (to a level of five and sevenfold over controls, respectively; Figure 2). The expression of the mRNAs for two other anti-oxidant enzymes (catalase and Cu/Zn superoxide dismutase [SOD1]) did not change significantly in most donors when examined by microarray or qPCR analysis (data not shown).

### Vitreous increases heme oxygenase-1 protein levels

**Figure 3:**
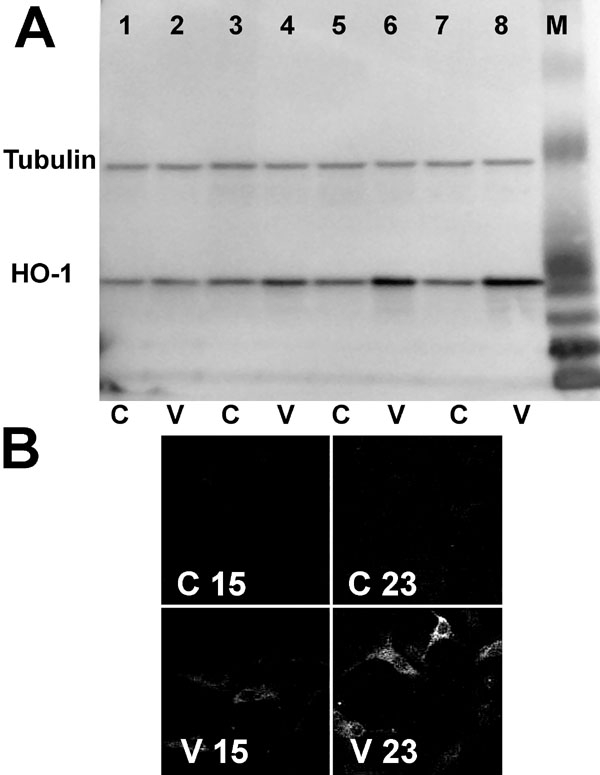
Expression of heme oxygenase-1 protein in vitreous-treated retinal pigment epithelial cells. **A**: Retinal pigment epithelial (RPE) cells were incubated with control medium (C) or vitreous-containing medium (V) for various times. The cells were washed, and proteins were extracted. The proteins were analyzed by immunoblotting using rabbit anti-heme oxygenase-1 or mouse anti-tubulin antibodies. Lanes 1 and 2: 3 h incubation. Lanes 3 and 4: 6 h incubation. Lanes 5 and 6: 12 h incubation. Lanes 7 and 8: 24 h incubation. M represents molecular weight markers. **B**: Cells were incubated with control or vitreous-containing medium for 15 (C15,V15) or 23 h (C23,V23). They were then fixed, permeabilized, and incubated with rabbit anti-HO-1 antibody followed by Texas red-conjugated goat anti-rabbit IgG.

An increase in HO-1 protein in the presence of vitreous after 6, 12, and 24 h (Figure 3A) was observed by immunoblotting. The absence of any detectable change in HO-1 at 3 h of treatment was consistent with the observation that vitreous has little effect on mRNA levels at this time point (Figure 1). A vitreous-induced increase in HO-1 protein was confirmed by immunofluorescence microscopy (Figure 3B).

### Transforming growth factor-β increases expression of heme oxygenase-1 but not metallothioneins

**Figure 4:**
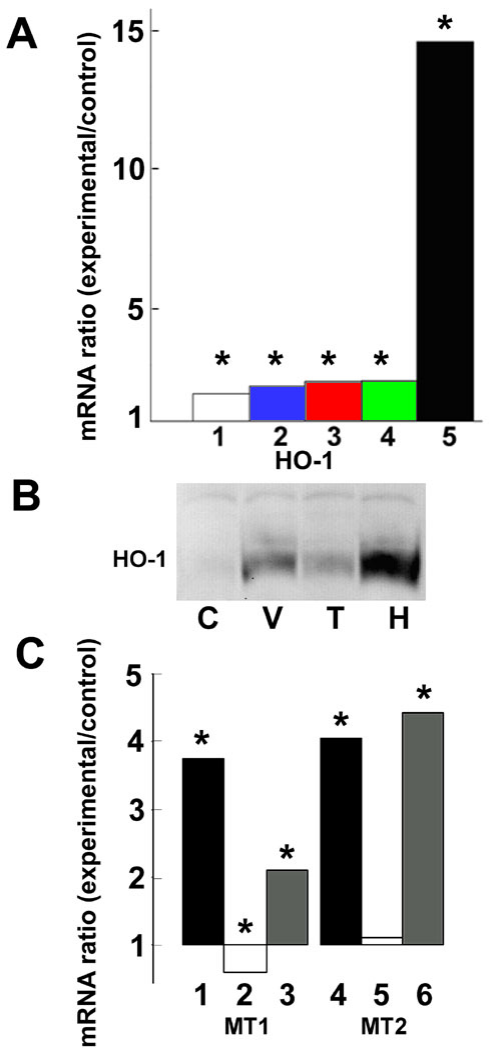
Effect of transforming growth factor-β1 on expression of heme oxygenase-1 and metallothioneins in retinal pigment epithelial cells. **A**: Retinal pigment epithelial (RPE) cells of the same donor and passage were incubated in control medium or with 25% vitreous or various concentrations (0.5 -10 ng/ml) of transforming growth factor-β1 (TGF-β1) for 12 h. Heme oxygenase-1 (HO-1) mRNA levels in vitreous- or TGF-β1-treated cells compared to control cells were measured by qPCR using ribosomal protein, large, P0 (RPLP0) mRNA levels to correct for any differences in the amount of cDNA. Bar 1: 0.5 ng TGF-β. Bar 2: 1 ng/ml. Bar 3: 5 ng/ml. Bar 4: 10 ng/ml. Bar 5: 25% vitreous. Values show the change in mRNA expression compared to control (no change =1). Asterisk (*) indicates that REST-XL analysis showed a change that is significantly different from control (p<0.05). **B**: RPE cells were incubated with normal medium (C), vitreous-containing medium for 24 h (V), TGF-β1 (5 ng/ml)-containing medium (T) for 24 h, or hemin-containing medium (H) for 24 h as a positive control. The proteins were analyzed by immunoblotting using rabbit anti-HO-1 antibodies. **C**: RPE cells were incubated with 25% vitreous (bars 1, 4) or 1 ng TGF-β1 per ml medium (bars 2, 5) or vitreous and TGF-β combined (bars 3, 6) for 12 h. mRNA for MT-1a (bars 1, 2, 3) and MT-2a (bars 4, 5, 6) in treated cells relative to untreated, control cells was determined. Asterisk (*) indicates that REST-XL analysis showed a change that is significantly different from control (p<0.05).

TGF-β upregulates anti-oxidant and anti-inflammatory proteins in human RPE [8] and other [28-30] cells. We therefore investigated whether it controls HO-1 and MT mRNA expression. Incubation of RPE cells with TGF-β1 increased the expression of HO-1 mRNA (Figure 4A). However, as the concentration of TGF-β1 was increased, the level of HO-1 mRNA expression reached a plateau that was usually much lower than that seen with 25% vitreous, suggesting that if TGF-β in vitreous were responsible for the effect on HO-1 mRNA expression, it was not the only factor. Immunoblotting also indicated that TGF-β1 induced HO-1 protein, but not to the extent shown by vitreous (Figure 4B). TGF-β1 did not increase expression of MT-1a or MT-2a mRNA when added to control medium and did not cause any increased expression compared to vitreous alone if added to medium containing vitreous (Figure 4C).

### Inhibitors of transforming growth factor-β signaling reduce vitreous-induced expression of heme oxygenase-1 mRNA

**Figure 5:**
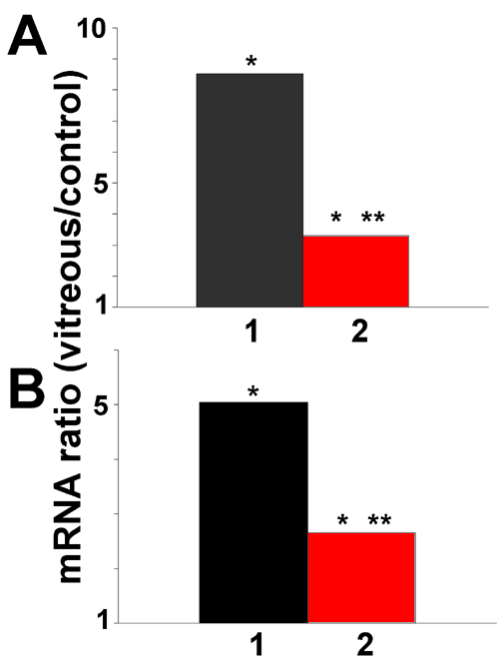
Inhibition of transforming growth factor-β signaling reduces the vitreous-mediated increase in heme oxygenase-1 expression by retinal pigment epithelial cells. **A**: Retinal pigment epithelial (RPE) cells were incubated for 12 h in vitreous-containing medium (bar 1) or in the same medium supplemented with SB431542 (10 μM), an inhibitor of type I TGF-β receptor signaling (bar 2). Heme oxygenase-1 (HO-1) mRNA expression was measured by qPCR. **B**: RPE cells were incubated in vitreous-containing medium (bar 1) or in the same medium supplemented with pan anti-transforming growth factor-β neutralizing antibody (10 μg/ml). HO-1 mRNA expression was measured by qPCR. Values show the change in mRNA expression compared to control (no change=1). Asterisk (*) indicates that REST-XL analysis showed a change that is significantly different from control levels of HO-1 mRNA (p<0.05). Double asterisk (**) denotes a value significantly different from the level of HO-1 mRNA in vitreous-treated cells (p<0.05).

To determine if TGF-β plays a role in the vitreous-induced increase in HO-1 mRNA expression, cells were incubated with vitreous, and TGF-β signaling was inhibited by addition of SB431542 (10 μM), a TGF-β type I receptor antagonist [25], or neutralizing pan-specific anti-TGF-β antibody. SB431542 inhibited the vitreous-induced expression of HO-1 mRNA by about 60% (Figure 5). Increasing the concentration of SB431542 did not increase the degree of inhibition of HO-1 expression (data not shown). Pan specific TGF-β antibody also caused a decrease in vitreous-stimulated HO-1 mRNA expression by about 50% (Figure 5). Neither SB431542 nor neutralizing TGF-β antibody affected MT-1a or MT-2a mRNA expression (data not shown).

### The vitreous-induced increase in heme oxygenase-1 mRNA expression but not metallothionein mRNA expression is mediated through reactive oxygen species generation

**Figure 6:**
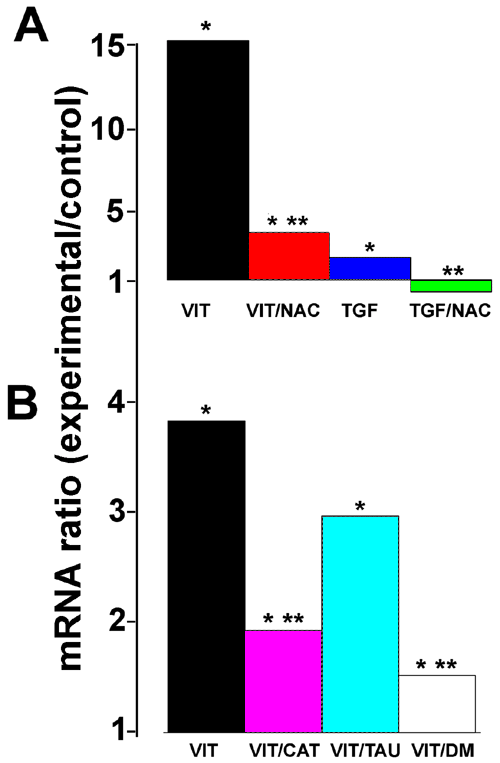
Reactive oxygen species quenchers inhibit the vitreous-mediated and transforming growth factor-β-mediated increase in heme oxygenase-1 mRNA expression. **A**: Retinal pigment epithelial (RPE) cells were incubated with control medium, vitreous-containing medium (VIT) or TGF-β1 (1 ng/ml)-containing medium for 12 h with or without 2 mM N-acetyl cysteine (NAC). HO-1 mRNA was measured by qPCR. All ratios are compared to the level in cells in control medium. **B**: RPE cells were incubated for 12 h in control medium, vitreous-containing medium (VIT) or vitreous-containing medium supplemented with 200 units catalase (VIT/CAT) per ml, 40 mM taurine (VIT/TAU) or 250 μM DMSO (VIT/DM). HO-1 mRNA was measured by qPCR. All ratios are relative to the level in cells in control medium (no change = ratio of 1.0). Asterisk (*) indicates that REST-XL analysis showed a change that is significantly different from control (p<0.05). Double asterisk (**) indicates a value significantly different from the level of HO-1 mRNA in vitreous-treated cells (p<0.05).

ROS have been implicated in upregulation of HO-1 and have been shown to be involved in some of the effects of TGF-β [31,32]. To determine whether TGF-β1- or vitreous-mediated upregulation of gene expression could involve ROS signaling, we incubated TGF-β1- or vitreous-treated RPE cells with or without N-acetyl cysteine (NAC), a glutathione analog that quenches ROS. The TGF-β-mediated rise in HO-1 mRNA expression was completely inhibited by NAC (Figure 6A). NAC also suppressed the vitreous-mediated rise in HO-1 mRNA expression, sometimes, though not always, to a level of 100% (Figure 6A).

**Figure 7:**
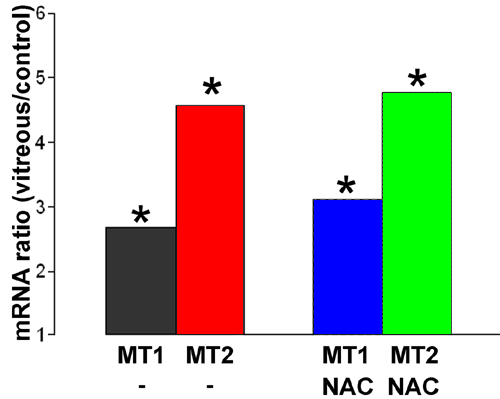
N-acetyl cysteine has no effect on the vitreous-mediated change in metallothionein expression. Cells were incubated with vitreous for 12 h with or without 2 mM N-acetyl cysteine (NAC). RNA was extracted and metallothionein-1 (MT-1) and metallothionein-2a (MT-2a) mRNA expression was measured by qPCR. Ratios show the change in mRNA expression compared to control (no change=ratio of 1). Asterisk (*) indicates that REST-XL analysis revealed a change that is significantly different from control (p<0.05).

NAC has been widely used to investigate whether ROS participate in a number of signaling pathways, including that initiated by TGF-β [33,34]. However, it has recently been shown that this inhibitor can act directly on TGF-β by reducing disulfide bonds, thereby inactivating it [35]. We therefore used several other quenchers of ROS (DMSO, taurine and catalase) that do not have the potential for disrupting disulfide bridges. Catalase and DMSO significantly inhibited the vitreous-mediated rise in HO-1 mRNA. The apparent inhibition by taurine was not statistically significant (Figure 6B). NAC had no effect on the vitreous-mediated rise in MT-1 or MT-2 expression (Figure 7).

### Vitreous induces heme oxygenase-1 mRNA expression via the activation of mitogen activated protein kinase pathways

MAPK pathways are frequently implicated in the signaling of HO-1 expression [7]. These consist of cascades of serine/threonine kinases, which sequentially phosphorylate and activate each other and target many transcription factors. The ERK, JNK, and p38 MAPK pathways have been intensively studied. JNK and p38 have been implicated in TGF-β signaling [36].

**Figure 8:**
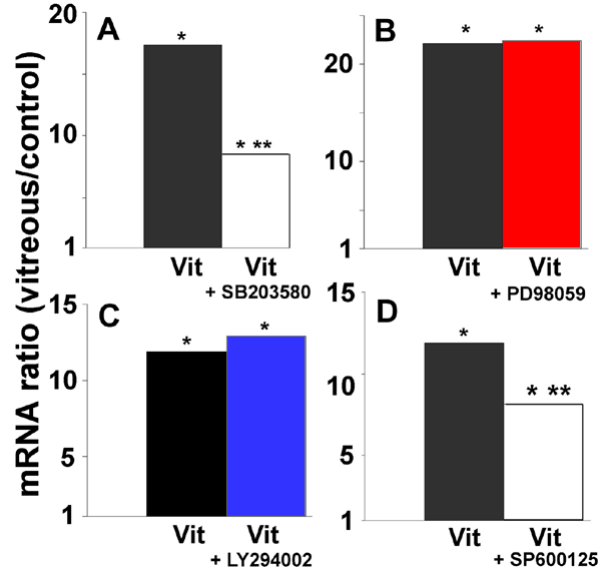
Inhibitors of p38 mitogen-activated protein kinase and Jun N-terminal kinase but not of extracellular signal-regulated kinase 1/2 or PI3 kinase inhibit the vitreous-mediated expression of heme oxygenase-1 mRNA. **A**: Cells were incubated for 12 h with control medium, 25% vitreous or 25% vitreous supplemented with 10 μM SB203580 which inhibits p38 mitogen-activated protein kinase (MAPK). RNA was extracted and heme oxygenase-1 (HO-1) mRNA was measured by qPCR. **B**: Cells were incubated for 12 h with control medium, 25% vitreous or 25% vitreous supplemented with 50 μM PD98059, which inhibits ERK1/2. **C**: Cells were incubated for 12 h with control medium, 25% vitreous or 25% vitreous supplemented with 50 μM LY294002, **D**: Cells were incubated for 12 h with control medium, 25% vitreous or 25% vitreous supplemented with 10 μM SP600125, which inhibits Jun N-terminal kinase (JNK). Ratios show the change in mRNA expression compared to control (no change equal to ratio of 1). Asterisk (*) indicates that REST-XL analysis showed a change that is significantly different from control levels of HO-1 mRNA (p<0.05). Double asterisk (**) denotes a value significantly different from the level of HO-1 mRNA in vitreous-treated cells (p<0.05).

Inhibition of the ERK pathway with PD98059 (50 μM) had no effect on the vitreous-mediated rise in HO-1 (Figure 8), MT-1a or MT-2a mRNAs (data not shown). Others have observed a lack of participation of this pathway in ROS-mediated upregulation of HO-1 expression [16]. As a positive control, we examined vitreous-induced expression of COX-2 mRNA and found that this was partly inhibited by PD98059 (data not shown). Inhibition of the PI3-kinase pathway by LY294002 (50 μM) also had no effect on the vitreous-induced increase in HO-1 mRNA (Figure 8) but, again, the vitreous-mediated increase in COX-2 expression was inhibited (data not shown). Addition of SB203580 (10 μM), which inhibits the α and β isoforms of p38 MAPK [37,38], resulted in approximately 50% inhibition of HO-1 mRNA induction. Inhibition of JNK by SP600125 (10 μM) also resulted in partial inhibition of HO-1 mRNA induction (Figure 8).

### Vitreous leads to the activation of AP-1/c-fos

Our results showed that vitreous increased HO-1 expression, at least in part, by TGF-β signaling and by ROS generation. The signaling pathways may also include p38 and JNK MAPKs, both of which have been implicated in TGF-β signaling [36]. TGF-β modulation of many genes is dependent on AP-1 binding sites in their promoters [39-41]. Such transcription factor binding sites have been reported in the promoters of human and mouse HO-1 genes; moreover, these sites can participate in ROS activation of HO-1 gene transcription [18,42-44]. Transcription factors that bind to AP-1 sites include c-jun/c-fos heterodimers [45] and these are potential targets of JNK and p38 MAPK pathways. We therefore investigated whether vitreous can activate AP-1 transcription factors, which might implicate them in the vitreous-mediated anti-oxidant response.

**Figure 9:**
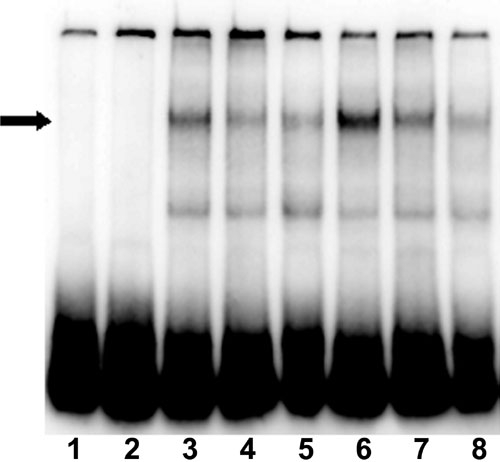
Vitreous activates AP-1 transcription factor. Nuclear protein extracts from control retinal pigment epithelial (RPE) cells or RPE cells treated with vitreous for one hour were incubated with a biotin-labeled probe, corresponding to the consensus binding sequence for AP-1. The probe-transcription factor complexes were separated from an unbound probe on a 5% polyacrylamide gel, transferred to nylon membranes, and detected by chemiluminescence. 1 represents labeled probe only; 2 represents labeled probe + anti-c-fos antibody; 3 represents control nuclear protein extract + labeled probe; 4 represents control nuclear protein extract + labeled probe + unlabeled probe; 5 represents control nuclear protein extract + labeled probe + anti-c-fos antibody; 6 represents vitreous-treated cell nuclear protein extract + labeled probe; 7 represents vitreous-treated cell nuclear protein extract + labeled probe + unlabeled probe; and 8 represents vitreous-treated cell nuclear protein extract + labeled probe + anti-c-fos antibody. The arrow indicates the position of the probe/AP-1 complex.

EMSA analyses showed that, compared to controls, nuclear extracts from RPE cells treated with vitreous for 1 h had increased protein binding activity for the AP-1 consensus sequence (arrow; Figure 9, compare lane 3 with lane 6). Addition of unlabeled AP-1 consensus sequence to the reaction decreased the intensity of the band formed by labeled AP-1 consensus sequence and AP-1 binding protein (arrow; Figure 9, lanes 4 and 7). To determine whether c-fos was part of the complex that bound to AP-1 binding sites after vitreous treatment, we carried out supershift assays. These indicated that c-fos was a component of the protein complex binding to the AP-1 consensus sequence. The protein-DNA band seen in the absence of c-fos antibody (Figure 9, lanes 3 and 6) was decreased in intensity when c-fos antibody was added (arrow; Figure 9, lanes 5 and 8), but a super-shifted DNA-protein-antibody band was not detected, possibly because the protein-DNA-antibody complex did not enter the gel.

### Vitreous increases c-fos mRNA and protein expression

**Figure 10:**
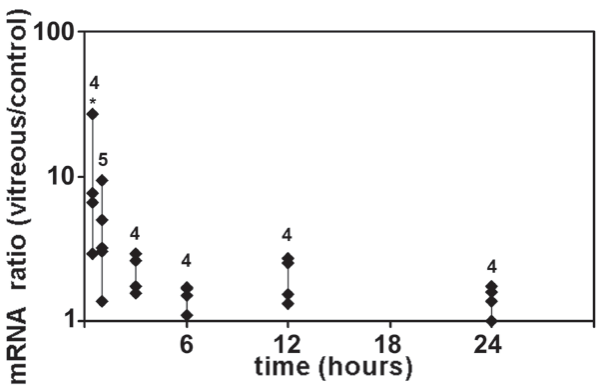
Vitreous increases c-fos mRNA levels in retinal pigment epithelial cells. Retinal pigment epithelial (RPE) cells were treated for various times with control medium or vitreous-containing medium, and the levels of mRNA for c-fos were measured using qPCR. Each point at a particular treatment time indicates a different RPE/vitreous donor pair, and the number of donor/vitreous pairs is shown. At 30 min, REST-XL analysis showed that c-fos mRNA was significantly increased (*p<0.004) in vitreous-treated RPE cells.

C-fos is an early response protein that is activated by phosphorylation [45]. Activation is sometimes accompanied by increased expression at the transcriptional level [46]. We used qPCR to determine the levels of c-fos mRNA after treatment with vitreous for various times. Multiple RPE cell and vitreous donors were used in these experiments. For all time points, cells were incubated with fresh complete medium or medium containing 25% vitreous for the same length of time. Compared to controls, significantly increased levels of c-fos mRNA (Figure 10, average = 11 fold, p<0.004) were found in RPE cells after 30 min of treatment with vitreous. After 1, 3, 6, 12, and 24 h of vitreous treatment, the levels of c-fos mRNA decreased compared to levels at 30 min and were not significantly higher than those of control cells.

**Figure 11:**
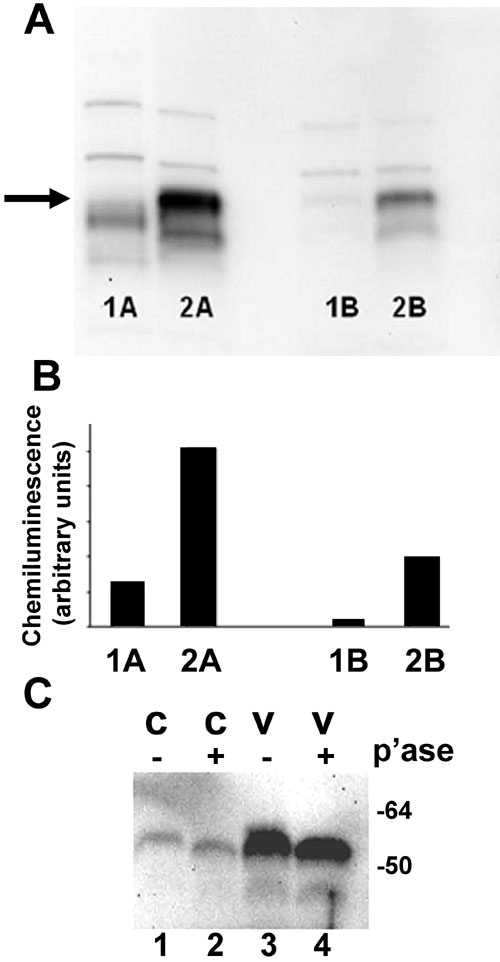
Vitreous increases c-fos protein expression and phosphorylation. **A**: Retinal pigment epithelial (RPE) cells were treated with 25% vitreous for one hour. Nuclear proteins were extracted and subjected to SDS-polyacrylamide gel electrophoresis and immunoblot analysis. Lanes 1A, 1B: Control nuclear protein extract. Lanes 2A, 2B: Nuclear protein extract from vitreous-treated RPE cells. Data shown are from two different RPE-vitreous donor pairs (**A** and **B**). The position of the c-fos band (62 kDa) is indicated by the arrow. **B**: Intensities of chemiluminescence corresponding to the bands at about 62 kDa in Figure 11A. A 4.3 fold (lane 2A compared to lane 1A) and 11.1 fold (lane 2B compared lane 1B) increase in c-fos protein was seen in the nuclear protein extracts of vitreous-treated RPE cells. **C**: RPE cells were treated with 25% vitreous for one hour. Nuclear proteins were extracted and treated with or without lambda protein phosphatase. Phosphatase (p'ase) treatment (+) resulted in the band at the position of c-fos moving faster in both control (lane 2) and vitreous-treated (lane 4) RPE nuclear protein extracts compared to nonphosphatase treated (-) samples (lanes 1, 3).

Nuclear protein extracts from control RPE cells or cells treated with vitreous for one hour were examined for expression of c-fos protein. Vitreous treatment resulted in increased expression (about four to fourteen fold) of c-fos protein, migrating at about 62 kDa, compared to the amounts of c-fos in the nuclear protein extracts from control cells (Figure 11A, arrow, and Figure 11B).

### C-fos protein is activated by phosphorylation in retinal pigment epithelial cells after vitreous treatment

Phosphorylation is one mechanism that increases the stability of c-fos protein [47]. Therefore, nuclear protein extracts from cells treated for 1 h with control medium or vitreous-containing medium were incubated with lambda protein phosphatase, which is active toward phosphorylated serine, threonine, and tyrosine residues. When electrophoretically separated, migration of c-fos in phosphatase-treated samples was faster indicating loss of phosphorylation (Figure 11C, compare lane 2 to lane 1 and lane 4 to lane 3). The c-fos present in nuclear protein extracts of both vitreous-treated and control RPE cells was found to be sensitive to phosphatase treatment (Figure 11C, lanes 2 and 4).

### C-fos protein moves into the nucleus on vitreous treatment

**Figure 12:**
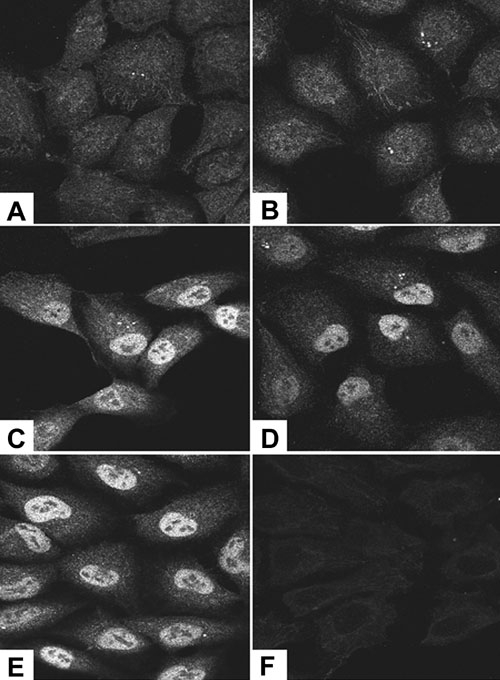
C-fos protein is increased in expression and moves into the nucleus of vitreous-treated retinal pigment epithelial cells. Retinal pigment epithelial (RPE) cells were treated with control medium or vitreous-containing medium for various periods of time. At 30 min, the control (**A**) and vitreous-treated (**B**) RPE cells had a similar pattern of staining for c-fos. The pattern of immunostaining remained the same for control cells at 1, 2, and 3 h (data not shown). Vitreous-treated RPE cells at 1, 2, and 3 h (**C**, **D**, **E**) showed intense staining for c-fos in the nucleus. **F**: Staining with secondary antibody only.

RPE cells were stained for c-fos protein after treating with control or vitreous-containing medium for various times and examined by immunofluorescence confocal microscopy. At 30 min, c-fos was visible in the nucleus and the cytoplasm. There was a small increase in nuclear c-fos in vitreous-treated (Figure 12B) compared to control RPE cells (Figure 12A).

The level of c-fos staining in control cells did not change after 1, 2, or 3 h treatment with fresh complete medium (data not shown). However, vitreous-treated RPE cells showed increased staining for c-fos at these time points, confirming increased expression at the protein level. In addition, the protein moved into the nucleus after 1 (Figure 12C), 2 (Figure 12D), or 3 (Figure 12E) h of treatment. Controls in which only secondary antibody was used did not show any staining (Figure 12F).

## Discussion

Vitreous has a profound influence on human RPE cells, causing them to acquire fibroblast-like characteristics in cell culture [48]. This EMT is similar to that which occurs in vivo in PVR [15,49]. Vitreous also stimulates RPE cells to express proteins associated with an inflammatory response; for example, the expression of IL-1β, COX-2, and mPGES is increased [21] (Ganti et al., In press). Increased oxidative stress is a frequent component of inflammatory disease, including in the eye [50], and subsequently provokes an anti-inflammatory, anti-oxidant response. Thus, proinflammatory cytokines such as TNF-α, upregulate antioxidant proteins, including HO-1 and MTs, as part of the resolution phase of inflammation [51]. Preinduction of HO-1 [52] or MTs [14] can ameliorate inflammation and protect against apoptosis and oxidative injury [12,51,53-59]. The observation that vitreous-treated RPE cells in culture express enhanced levels of HO-1, MT-1a, and MT-2a is consistent with these proteins being induced as part of a protective response against stress and inflammatory mediators.

There are several isoforms of HO. HO-1 is the inducible form [7]. This enzyme catalyzes the breakdown of heme to iron, biliverdin, and carbon monoxide. Biliverdin is then reduced to bilirubin (a powerful anti-oxidant) while iron (a pro-oxidant) is sequestered by ferritin [7]. Several genes encode MTs in a tissue-specific manner but most commonly expressed are MT1 and MT2 of which there are several isoforms [54]. These small multifunctional cysteine-rich proteins act as anti-oxidants by scavenging oxygen and hydroxyl radicals and as anti-apoptotic agents by inhibiting caspase-3 activation [54]. HO-1 expression is elevated during inflammation resulting from the expression of proinflammatory cytokines (including TNF-α, interleukins 1α, 1β, 6, 8, 12, and 18 and interferon-γ) [60-62] and many of the same cytokines also up regulate MT expression [14,63]. Subsequently, carbon monoxide, a product of HO-1 activity, suppresses the synthesis of proinflammatory cytokines, nitric oxide, and prostaglandins [55-57] and the use of MT knockout mice suggests a similar function for MTs [58,59]. Thus, these proteins provide a negative feedback during inflammation that inhibits oxidative damage [53].

The importance of HO-1 and MT as anti-inflammatory agents led us to investigate the components of vitreous that may control anti-oxidant gene expression. Although vitreous comprises mainly collagen and water, it contains a number of other proteins in smaller amounts, among which are many growth factors. Many of these vitreous constituents are not well characterized, and it is unlikely that a single stimulatory molecule is responsible for the induction of HO-1 and MTs; more probable is a complex interaction between multiple factors that act synergistically to promote an anti-oxidant response. One well characterized vitreous protein, however, is TGF-β, which has been implicated in PVR [15] and in EMT responses in general [64]. This growth factor induces HO-1 in RPE cells [8] and many other cell types [7]. Thus, we examined the role of TGF-β in the anti-oxidant response using inhibitors of TGF-β signaling.

When TGF-β binds to the TGF-β receptor (TGF-βR), the TGF-βR type II component phosphorylates the TGF-βR type I component, activating its kinase activity and leading to further phosphorylation signaling events, including the activation of smad 2 and 3 transcription factors [65]. SB431542 is a TGF-βR type I antagonist [25], which substantially inhibits vitreous-mediated upregulation of HO-1, as does a panspecific anti-TGF-β neutralizing antibody. This implies that a TGF-β-dependent pathway plays an important role in the induction of HO-1. The fact that neither inhibitor reduces the increase in expression of HO-1 by more than 60% suggests that other pathways are also involved. This is not surprising since HO-1 mRNA expression can be controlled by multiple pathways at both the transcriptional and post-transcriptional levels [7]. Neither SB431542 nor the neutralizing antibody had any effect on vitreous-mediated MT1a or MT2a mRNA expression and thus vitreous factors other than TGF-β are responsible for MT mRNA induction. Moreover, TGF-β added to RPE cells, while inducing HO-1, had no effect on MT mRNA expression as measured by qPCR, although we previously found a weak response in RPE cells measured by Northern blotting [14]. TGF-β added to RPE cells did not induce HO-1 to the level that would have been expected, given the effect of TGF-β inhibitors on vitreous-induction of HO-1. This could be because there are factors in vitreous that act synergistically to increase signaling via the TGF-β-dependent pathway.

HO-1 gene expression is frequently induced by ROS [7] and TGF-β can signal via ROS generation [66-68]. It was therefore possible that vitreous could induce changes in expression of HO-1 genes via ROS generation. Several ROS quenchers decreased vitreous-mediated HO-1 mRNA expression. NAC is a glutathione analog and ROS scavenger and usually completely suppressed HO-1 upregulation by vitreous or TGF-β, although it had little, if any, effect on MT-1a or MT-2a mRNAs. Although NAC has been commonly used to implicate ROS in signaling pathways [69], it has other effects that complicate the interpretation of the data. For example, it can act directly on TGF-β leading to its inactivation [35]. Thus, the inhibition of the vitreous effect could be due to inhibition of the TGF-β-dependent component as well as a ROS-dependent component. Because of this, other ROS scavengers and quenchers were used, including catalase (which breaks down hydrogen peroxide), taurine [70], and DMSO [71]. All of these reduced vitreous-mediated induction of HO-1 expression and supported the importance of the signaling role of ROS, although none of them showed as great an effect as NAC; this would be consistent with NAC suppressing other pathway(s) besides those mediated via ROS.

TGF-β classically signals via smads, which bind to the promoters of their target genes [72]. However, other signaling pathways via ERK, p38 MAPK, and JNK are also important [36,41]. Although these MAPK pathways often transmit growth factor, extracellular matrix and cytokine signals, respectively, there is cross-talk between them that is particularly evident between the p38 and JNK pathways [73]. These signaling pathways participate in the control of HO-1 expression in a number of cell systems [7]. In human RPE cells, the PI3 kinase and MEK 44/42 pathways appear not to be involved in the control of HO-1 expression by vitreous, as judged by the lack of effect of LY294002 and PD98059, respectively, whereas inhibitors of the JNK and the p38MAPK pathways (SP600125 and SB203580) reduced vitreous-meditated HO-1 mRNA expression. P38 MAPK can be activated by TGF-β [74-76] and mediates some of its effects in a smad-independent [75], AP-1-dependent manner [76,77]. JNK can also be activated by TGF-β [76] and regulates the activity of AP-1 transcription factor complexes [78] (as well as smads [79]) by phosphorylation. These data suggest the possibility that vitreous and TGF-β might induce their anti-oxidant responses via AP-1 activation.

AP-1 complexes consist of homo- or hetero dimers of basic leucine zipper family members, including c-jun, c-fos, and several other proteins [80,81]. We found vitreous caused an increase in AP-1 site binding activity, and that part of this was associated with complexes containing c-fos. Vitreous also caused an increase in c-fos mRNA and protein expression and translocation of c-fos to the nucleus. This is important because AP-1 activity has been implicated in expression of HO-1 and MTs. The promoter region of the HO-1 gene has many transcription factor binding sites, including those for AP-1, leading to complex regulation [7,82]. MT-1 [83] and MT-2 [84-86] promoters also contain AP-1 binding sites. Studies of enhanced HO-1 and MT expression as a result of oxidative stress and inflammation often implicate AP-1 transcription factor activation [16,44,87] and, although the promoter elements that control TGF-β-mediated HO-1 induction have not been identified, AP-1 activation has been implicated in the induction of several other TGF-β-responsive genes [88,89].

In summary, our data show that vitreous can initiate an anti-oxidant response in human RPE cells, as exemplified by an increase in HO-1 and MT expression. It is likely that TGF-β and ROS signaling participate in the induction of HO-1 by vitreous, and that the p38 MAPK and JNK pathways are involved. Both p38 and JNK target AP-1 transcription factor complexes and, since AP-1 is important to the regulation of HO-1 and MT in other systems, it is possible it is involved in the vitreous-mediated response. In contrast, the pathway for MT induction by vitreous is quite different and does not utilize TGF-β or ROS signaling and does not involve p38 or JNK signaling.
